# Plasma Metabolic Profiles in Women are Menopause Dependent

**DOI:** 10.1371/journal.pone.0141743

**Published:** 2015-11-18

**Authors:** Chaofu Ke, Yan Hou, Haiyu Zhang, Kai Yang, Jingtao Wang, Bing Guo, Fan Zhang, Hailong Li, Xiaohua Zhou, Ying Li, Kang Li

**Affiliations:** 1 Department of Epidemiology and Biostatistics, School of Public Health, Harbin Medical University, Harbin, 150086, P.R. China; 2 Department of Biostatistics, School of Public Health and Community Medicine, University of Washington, Seattle, United States of America; 3 Department of Nutrition and Food Hygiene, School of Public Health, Harbin Medical University, Harbin, 150086, P.R. China; Korea University, REPUBLIC OF KOREA

## Abstract

Menopause is an endocrinological transition that greatly affects health and disease susceptibility in middle-aged and elderly women. To gain new insights into the metabolic process of menopause, plasma metabolic profiles in 115 pre- and post-menopausal women were systematically analyzed by ultra-performance liquid chromatography/mass spectrometry in conjunction with univariate and multivariate statistical analysis. Metabolic signatures revealed considerable differences between pre- and post-menopausal women, and clear separations were observed between the groups in partial least-squares discriminant analysis score plots. In total, 28 metabolites were identified as potential metabolite markers for menopause, including up-regulated acylcarnitines, fatty acids, lysophosphatidylcholines, lysophosphatidylethanolamines, and down-regulated pregnanediol-3-glucuronide, dehydroepiandrosterone sulfate, *p*-hydroxyphenylacetic acid and dihydrolipoic acid. These differences highlight that significant alterations occur in fatty acid *β*-oxidation, phospholipid metabolism, hormone metabolism and amino acid metabolism in post-menopausal women. In conclusion, our plasma metabolomics study provides novel understanding of the metabolic profiles related to menopause, and will be useful for investigating menopause-related diseases and assessing metabolomic confounding factors.

## Introduction

Menopause usually occurs naturally in women during their late 40s or early 50s, and signals cessation of a woman's reproductive ability. As the human life expectancy has increased dramatically, women are expected to spend more than a third of their lifetime in menopause.[1] As we know, menopause was often associated with hot flashes, joint pain, muscle pain, depressed mood and sleep disturbances.[2] Most importantly, post-menopausal women are also at a high risk of developing various diseases such as cardiovascular diseases (CVDs) and osteoporosis.[3] It has been suggested that the incidence of CVDs increases sharply after the occurrence of menopause in women.[4] Thus, understanding the physiology of menopause is of great significance to allow women to grow disease-free and with a good quality of life.

The field of metabolomics is an emerging field dedicated to the global quantitative measurement of endogenous small molecule metabolites within a biological system.[5] Recently, it has been successfully used to build metabolic profiles associated with biological activities, physiological status and disease history.[6–12] Applying a NMR-based metabolomic profiling method, Zhang *et al*. found strong associations between the metabolome and gender, pubertal development, and physical activity in overweight adolescents.[6] Yu *et al*. reported that human serum metabolic profiles are age dependent and might reflect different aging processes.[7] Our previous plasma and urinary metabolomic studies have also identified novel biomarkers and metabolic dysregulations for ovarian cancer.[10–12] These findings indicate that metabolic profiling could provide a new approach to explore the metabolic effects of various conditions in complex biological systems. To date, menopause-associated metabolomic studies have been quite limited.

In this study, we performed plasma metabolic profiling of plasma from 52 pre-menopausal women and 63 post-menopausal women using an ultra-performance liquid chromatography mass spectrometry (UPLC/MS) platform. Menopause-related metabolic signatures were explored with electrospray ionization (ESI) in both positive ion mode and negative ion mode. The aim was to discover potential biomarkers for menopause and gain new insights into the metabolic process of menopause.

## Materials and Methods

### Pre- and post-menopausal women

This study was approved by the Ethics Committee of Harbin Medical University. One hundred and fifteen women aged 39 to 66 years from the Daoli district in Harbin, China were recruited with informed written consents. None of the participants had any metabolic diseases, liver/renal diseases or inflammatory diseases when entering the current study. Menstrual history questionnaires were used to obtain information on menopausal status. Fifty-two women aged 39 to 52 years reported regular menstruation without hormonal agents and were deemed as pre-menopausal, and 63 women aged 46 to 66 years reported amenorrhea for more than 12 months and were defined as post-menopausal. After fasting for 12h, 5 mL of whole blood was collected by venipuncture from each participant. The fresh blood samples were centrifuged at 1,000×*g* for 10 min and the supernatant was extracted and frozen at –80°C.

### Sample pretreatment

The plasma samples were thawed in a 4°C refrigerator for 40 min. A 200 μL aliquot of each plasma sample was mixed with acetonitrile (3×200 μL) and vortexed for 1 min. After mixing, the solutions were centrifuged at 12,000 ×*g* for 15 min at 4°C. An aliquot (300 μL) of each supernatant was transferred into a clear vial and dried in a vacuum rotary dryer, and the residue was dissolved in 100 μL of acetonitrile/water (1:3, v/v). The solutions were vortexed for 5 min, and then centrifuged at 12,000 ×*g* for 15 min at 4°C. The supernatants were then placed into sample vials for analysis. Before UPLC/MS analysis, all the samples were randomized. The pooled quality control (QC) samples were prepared by mixing equal amounts of plasma samples from 5 pre-menopausal subjects and 5 post-menopausal subjects. One QC sample was run every 10 samples.

### Chromatography

A 10 μL aliquot of the pretreated sample was injected into an ACQUITY UPLC BEH C18 column (2.1 × 100mm, 1.7μm; Waters Corporation, Milford, MA) and analyzed using an UPLC system (Waters Corporation). The UPLC mobile phase consisted of acetonitrile containing 0.1% formic acid (solvent A) and water containing 0.1% formic acid (solvent B). The flow rate was set at 0.3 mL/min. A linear mobile phase gradient was used as follows: 1% A, held for 0.5 min; 0.5–4.0 min, increased from 1 to 15% A; 4.0–4.5 min, increased from 15 to 55% A; 4.5–11.5 min, increased from 55 to 90% A; 11.5–12.0 min, increased from 90 to 99% A; and held at 99% A from 12.0–15.0 min. After the analytical run, the mobile phase was returned to 1% A in 0.1 min and equilibrated at 1% A for 1 min.

### Mass spectrometry

Mass spectrometry was performed on an Agilent 6520-QTOF (Agilent Technologies) operating at both positive-ion (ESI+) and negative-ion (ESI–) electrospray ionization (ESI) modes. The capillary voltage was 4.0 kV in ESI+ mode and 3.5 kV in ESI- mode. The desolvation temperature was 330°C, the gas flow rate was set at 10 L/min, and nitrogen was used as the drying gas. Centroid data were collected in the full scan mode from 50 to 1000 *m/z*.

### Data preprocessing and annotation

The raw UPLC-QTOF/MS data were first converted into mzData-format files by MassHunter Qualitative Analysis Software (Agilent Technologies). Then these files were imported to the XCMS package in the R platform for preprocessing, which included peak detection, peak matching and retention time alignment.[13] The following XCMS parameters were utilized: xcmsSet (method = "centWave", peakwidth = c(5, 20)); group (bw = 10); retcor (method = "obiwarp"). The other parameter settings were as default. The CAMERA package in R was further used for annotation of isotope peaks, adducts and fragments.[14]

### Statistical analysis

Principal component analysis (PCA) and partial least-squares discriminant analysis (PLS-DA) were performed on SIMCA-P 11.5 software. Welch’s *t*-test, heat map and false discovery rate (FDR) were performed using R. PCA was first used to detect grouping trends and outliers.[15] PLS-DA was then performed to understand global metabolic changes between pre-menopausal women and post-menopausal women.[15] To avoid overfitting, permutation tests with 100 iterations were performed to validate the supervised model.[16] Variable importance in the projection (VIP) for each metabolite was calculated based on the established PLS-DA model. In addition, the Welch’s *t* test was performed to determine the significance of each metabolite, and the corresponding FDR value was estimated for correcting multiple comparisons.[17] To visualize the metabolite changes by menopause, the standardized metabolite level in 115 women was plotted in a heat map.

## Results

Details about demographic and clinical characteristics of the participants can be referred to Table 1. After isotope peaks were excluded, 2048 ions in ESI+ mode and 1757 ions in ESI– mode were used for statistical analysis. The PCA performed on all the samples revealed that the QC samples were tightly clustered in the PCA score plots (S1 Fig), suggesting satisfactory stability and repeatability of the sample analysis sequence.

**Table 1:** Demographic and clinical characteristics of the participants.

Characteristics	Pre-menopausal women (n = 52)	Post-menopausal women (n = 63)	P value
Age (year)	45.77±3.23	56.38±4.53	<0.0001 ^b^
BMI	24.53±3.10	24.44±2.92	0.8695 ^a^
Fasting blood glucose (mmol/L)	4.09±0.49	4.25±0.58	0.1268 ^a^
High density lipoprotein(mmol/L)	1.25±0.29	1.25±0.31	0.9904 ^a^
Low density lipoprotein (mmol/L)	2.65±0.61	3.09±0.78	0.0014 ^a^
Triglyceride (mmol/L)	1.10 (0.70~1.68)	1.28 (0.94~1.82)	0.0844 ^c^
Total cholesterol (mmol/L)	4.64 (4.17~5.00)	5.22 (4.73~5.83)	0.0002 ^c^
Systolic pressure (mmHg)	120.50 (115.00~129.00)	124.00 (119.00~143.00)	0.0813 ^c^
Diastolic pressure (mmHg)	79.00(72.00~85.00)	77.00 (74.00~84.00)	0.6280 ^c^

Note: If the data are normally distributed, description of mean±standard deviation, Student's *t* test (equal variance) ^a^ or Welch's *t* test (unequal variance) ^b^ were used. If the data are not normally distributed, description of median (interquartile range) and Wilcoxon rank sum test ^c^ were used.

**Fig 1:**
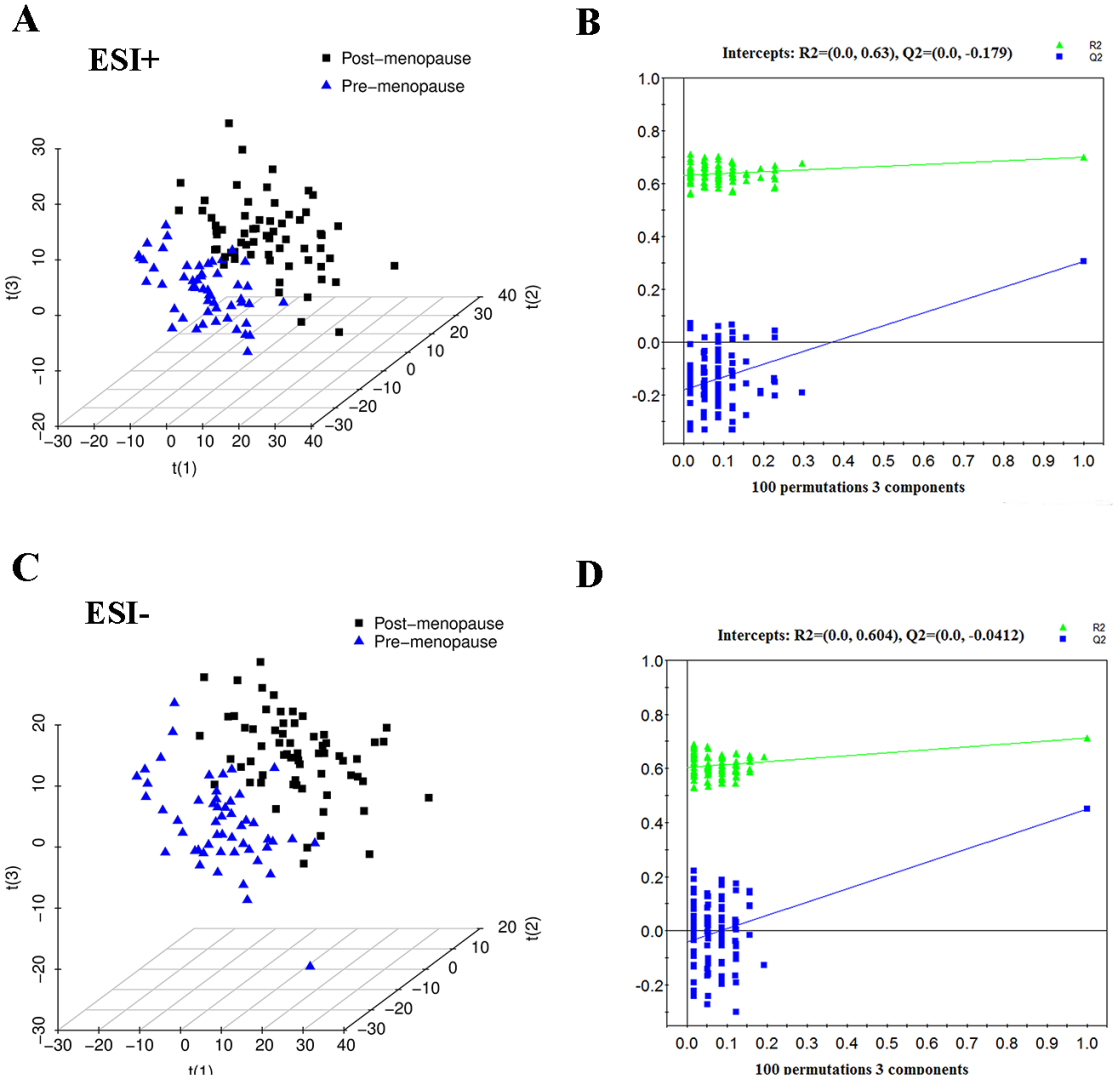
PLS-DA three-dimensional scores plots and validation plots. (A) PLS-DA three-dimensional scores plot for pre-menopausal women *versus* post-menopausal women in ESI+ mode (three latent variables, R2X = 0.208, R2Y = 0.701, Q2 = 0.306). (B) Validation plot for pre-menopausal women *versus* post-menopausal women in ESI+ mode. (C) PLS-DA three-dimensional scores plot for pre-menopausal women *versus* post-menopausal women in ESI- mode (three latent variables, R2X = 0.278, R2Y = 0.713, Q2 = 0.450). (D) Validation plot for pre-menopausal women *versus* post-menopausal women in ESI- mode.

Although unsupervised PCA only showed separation trends between pre- and post-menopausal women (S1 Fig), clear discriminations of pre-menopausal women from post-menopausal women were observed in the PLS-DA score plots (Fig 1A and 1C). Both of the PLS-DA models contained three latent variables with the performance values of R2X = 0.208, R2Y = 0.701, Q2 = 0.306 in ESI+ mode and R2X = 0.278, R2Y = 0.713, Q2 = 0.450 in ESI- mode. The validation plots assured the validity and robustness of all the PLS-DA models (Fig 1B and 1D), as all permuted R2 and Q2 values on the left were lower than the original point on the right, and the Q2 regression line in blue had a negative intercept.[18] Additional permutation tests based on 2000 iterations was used to obtain robust p values (ESI+: P<0.0005; ESI-: P<0.0005) for the PLS-DA models (S2 Fig).

**Fig 2:**
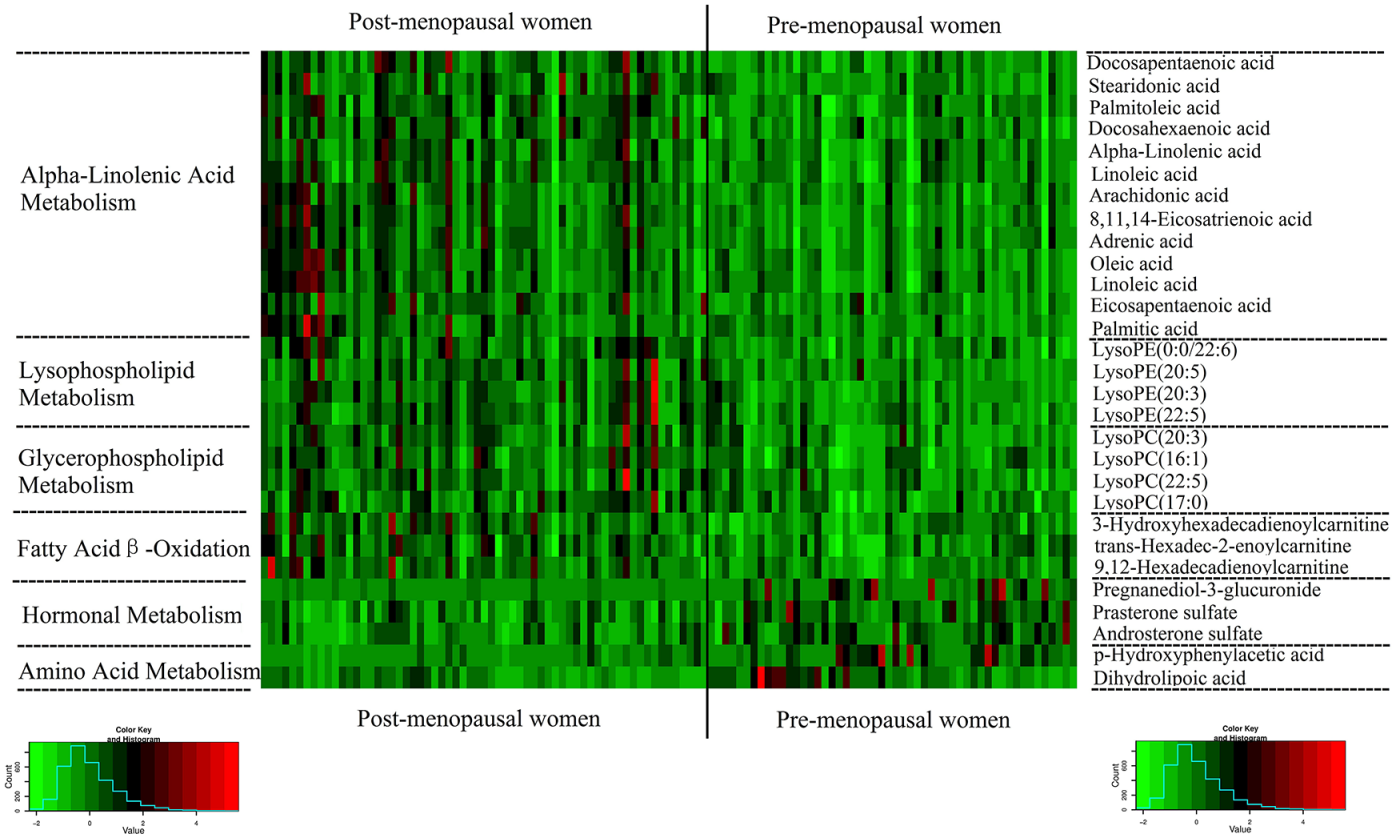
Heat map demonstrating 28 menopause-related metabolites in 115 pre-menopausal and post-menopausal women.

Analysis of variable importance project (VIP) values revealed discriminatory metabolites that contributed to the classification of pre- and post-menopausal women. Based on FDR values and VIP thresholds of 0.05 and 1, respectively, differential ions were selected as biomarker candidates for subsequent metabolite identification. The identification procedures were similar to our previously published strategies.[9, 10] In total, 16 metabolites in ESI+ mode and 12 metabolites in ESI– mode were identified (Table 2). Linoleic acid, arachidonic acid and prasterone sulfate were further verified by standard references. Compared with pre-menopausal women, lysophosphatidylcholines (LPCs), lysophosphatidylethanolamines (LPEs), unsaturated and saturated fatty acids, and acylcartinines levels were observed to be elevated in post-menopausal women. By contrast, dehydroepiandrosterone sulfate (DHEAS), androsterone sulfate, pregnanediol-3-glucuronide, p- hydroxyphenylacetic acid, and dihydrolipoic acid levels were decreased in post-menopausal women compared to pre-menopausal women (Fig 2). The involved biochemical pathways mapped in HMDB [19] and KEGG [20] included fatty acid β-oxidation, lysophospholipid metabolism, glycerophospholipid metabolism, hormone metabolism and amino acid metabolism.

**Table 2:** Twenty-eight potential metabolic biomarkers identified for menopause.

Metabolite	Mode	m/z	RT	P value^a^	FDR	VIP	FC^b^	pathway
Docosapentaenoic acid	ESI+	331.2632	13.28	5.70E-06	2.98E-03	1.84	0.64	Alpha Linolenic Acid Metabolism
Stearidonic acid	ESI+	277.2156	11.67	2.05E-05	4.51E-03	1.58	0.55	Alpha Linolenic Acid Metabolism
Palmitoleic acid	ESI+	255.2319	13.05	3.68E-05	4.96E-03	1.90	0.54	Alpha Linolenic Acid Metabolism
Docosahexaenoic acid	ESI+	329.2476	12.91	1.62E-05	4.27E-03	1.84	0.47	Alpha Linolenic Acid Metabolism
Alpha-Linolenic acid	ESI+	279.2318	12.52	2.06E-03	3.19E-02	1.70	0.35	Alpha Linolenic Acid Metabolism
LysoPE(0:0/22:6)	ESI+	526.2932	8.93	8.71E-06	3.52E-03	1.68	0.33	Lysophospholipid metabolism
LysoPE(20:5)	ESI+	500.2775	8.20	5.08E-05	5.94E-03	1.64	0.52	Lysophospholipid metabolism
LysoPE(20:3)	ESI+	504.3087	9.67	2.89E-05	4.79E-03	1.59	0.49	Lysophospholipid metabolism
LysoPE(22:5)	ESI+	528.3091	9.38	3.47E-04	1.57E-02	1.35	0.46	Lysophospholipid metabolism
LysoPC(20:3)	ESI+	546.3557	9.73	4.31E-04	1.66E-02	1.34	0.38	Glycerophospholipid metabolism
LysoPC(16:1)	ESI+	494.3243	8.52	9.44E-04	2.41E-02	1.28	0.31	Glycerophospholipid metabolism
LysoPC(22:5)	ESI+	570.3551	9.43	3.42E-03	4.12E-02	1.14	0.31	Glycerophospholipid metabolism
LysoPC(17:0)	ESI+	510.3558	10.84	4.19E-04	1.65E-02	1.58	0.24	Glycerophospholipid metabolism
3-Hydroxyhexadecadienoylcarnitine	ESI+	412.3051	6.93	8.42E-05	8.31E-03	1.54	0.38	Fatty acid β-oxidation
trans-Hexadec-2-enoylcarnitine	ESI+	398.3264	8.50	1.65E-03	2.92E-02	1.33	0.26	Fatty acid β-oxidation
9,12-Hexadecadi-enoylcarnitine	ESI+	396.3103	7.76	1.89E-04	1.26E-02	1.45	0.39	Fatty acid β-oxidation
Arachidonic acid	ESI-	303.2334	13.10	2.89E-05	1.74E-03	1.45	0.57	Alpha Linolenic Acid Metabolism
8,11,14-Eicosatrienoic acid	ESI-	305.2488	13.60	2.60E-06	7.27E-04	1.63	0.60	Alpha Linolenic Acid Metabolism
Adrenic acid	ESI-	331.2644	13.83	7.61E-05	2.43E-03	1.55	0.56	Alpha Linolenic Acid Metabolism
Oleic acid	ESI-	281.2496	14.02	5.72E-05	2.09E-03	1.43	0.65	Alpha Linolenic Acid Metabolism
Linoleic acid	ESI-	279.2341	13.30	2.16E-04	4.08E-03	1.42	0.50	Alpha Linolenic Acid Metabolism
Eicosapentaenoic acid	ESI-	301.2176	12.32	6.61E-05	2.26E-03	1.36	0.73	Alpha Linolenic Acid Metabolism
Palmitic acid	ESI-	255.2335	13.95	8.43E-04	7.39E-03	1.34	0.55	Alpha Linolenic Acid Metabolism
Pregnanediol-3-glucuronide	ESI-	495.2962	6.54	8.66E-05	2.58E-03	2.09	-1.18	Pregnanediol metabolism
Prasterone sulfate	ESI-	367.1597	6.95	6.68E-04	6.65E-03	1.50	-0.59	Steroid hormone biosynthesis
Androsterone sulfate	ESI-	369.1734	7.21	5.63E-04	6.21E-03	1.57	-0.93	Steroid hormone biosynthesis
p-Hydroxyphenylacetic acid	ESI-	151.0402	6.20	1.18E-05	2.94E-03	1.92	-1.37	Tyrosine metabolism
Dihydrolipoic acid	ESI-	207.0512	1.75	1.35E-03	9.72E-03	1.88	-0.54	Glycine and Serine Metabolism

Abbreviations: Electrospray ionization (ESI); False discovery rate (FDR); Fold change (FC); Measured mass to charge ratio (m/z); Retention time (min, RT); Variable importance in the projection (VIP);

^a^ P value was obtained by the Welch’s *t* test;

^b^ Fold change (FC) was calculated from the arithmetic mean values of each group. Fold change with a positive value indicates that the concentration of certain metabolite is up-regulated in post-menopausal individuals compared to pre-menopausal individuals.

## Discussion

Menopause has significant effects on a number of organ systems including the cardiovascular, skeletal, endocrinological and central nervous systems. These effects could induce a series of physiological and potential pathological changes. Establishing menopause-related metabolic signatures will facilitate our understanding of these influences, and have important biological implications. In this study, untargeted metabolic profiling was performed to investigate metabolic signatures associated with menopause. Significant metabolic differences were detected between pre- and post-menopausal women, and 28 metabolites were finally identified as potential biomarkers for menopause.

### A metabolic view on menopause

Previous metabolomic studies on menopause have observed evident relationships between menopause and metabolic changes.[8, 21] Auro *et al*. utilized a NMR-based platform to investigate the metabolism of menopause at the population level and found that menopause status was associated with total, monounsaturated, omega-7 and -9 fatty acids and several amino acids.[8] However, this study only targeted 135 molecules, of which, 74 were macromolecular lipoproteins. Yamatani *et al*. also reported that levels of fatty acid metabolites were significantly higher in the visceral fat of post-menopausal women than in premenopausal women.[21] In addition to fatty acid metabolism, our study look at lipid profiles in more details and at hormone metabolism and amino acid metabolism at a small-molecular level, adding to the current understanding of menopause-induced metabolic changes.

The most remarkable changes in post-menopausal women were characterized by significantly increased lipid-related metabolites, including LPCs, LPEs, acylcarnitines, and fatty acids. Post-menopausal women are known to be highly susceptible to these diseases. Studies have shown that LPC acts synergistically with endothelin-1 in inducing vascular smooth muscle cell proliferation.[22] Accumulation of fatty acid intermediates in the cardiomyocyte cytosol could result in lipotoxicity and increase cardiovascular risk in menopausal women.[23] In addition, studies have demonstrated that products of lipid and lipoprotein oxidation may contribute to reduced bone mass and bone fragility.[24, 25] An increasing body of biological and epidemiological evidence has also provided support for a link between CVDs and osteoporosis.[26] From this point of view, lipid metabolism might be a one of the common mechanisms underlying the pathogenesis of both CVD and osteoporosis. After all, the significant changes observed in lipid-related metabolites in postmenopausal women certainly warrant further investigation with respect to chronic diseases such as CVDs and osteoporosis.

Significantly decreased concentrations of pregnanediol-3-glucuronide, androsterone sulfate and DHEAS were detected in post-menopausal women. These compounds are all hormone metabolites or precursors,[19] and the changes observed in them indicated a significant drop of hormones after menopause. Hormonal changes could lead to a cascade of physiological and pathological changes, which could account for many phenomena and diseases associated with menopause (e.g. osteoporosis).[27] Studies have also shown that DHEAS levels are inversely associated with mortality of coronary artery diseases,[28] although the underlying mechanisms have yet to be determined. In addition, two amino acid metabolites, *p*-hydroxyphenylacetic acid and dihydrolipoic acid, were found down-regulated in post-menopausal women. In recent years, amino acid changes have been linked to increased risk of diabetes and CVDs.[29] It has been suggested that dihydrolipoic acid has antioxidant activity against microsomal lipid peroxidation, which contributes to atherosclerosis.[19] The exact roles of these metabolic changes in menopause-related diseases deserve our attention and await further investigation.

### Menopause as a potential confounder in metabolomics

Over the last decade, the number of research articles published in the field of metabolomics has increased dramatically.[30] Compared with genomics or proteomics, metabolomics can reflect changes in biological phenotypes more directly and immediately.[5] However, metabolic changes can be attributed to a variety of factors, including both biological sources of interest and uncontrolled confounding sources. Our study demonstrated that menopause could be an important contributor to the metabolic profile of body fluids, which makes menopause as a possible confounder in metabolomic studies. Many metabolomic studies have used designs that do not account for the effects of menopause.[10, 31–33] Remarkably, some of these studies have identified biomarkers that overlapped with menopause-related biomarkers identified in the present study.[10, 31, 32] In this case, it is difficult to distinguish whether these biomarkers resulted from biological sources of interest or uncontrolled menopause status, and these biomarkers require verification. In future studies, menopause should be considered and balanced in metabolomics designs. Alternatively, menopause should be at least adjusted for in statistical analysis to avoid any false discoveries. This would increase the reliability and repeatability of metabolomic studies.

In this study, total cholesterol (TC) and low density lipoprotein (LDL) were imbalanced between pre-menopausal women and post-menopausal women. However, all the biomarkers remained statistically significant after adjusting for TC and LDL in logistic regression models. In addition, the widely-used acetonitril extraction would restrict lipid profiling to more hydrophilic lipids, which prevented a more comprehensive understanding of lipid metabolism of menopause. Lipidomics would be needed to validate and complement current findings.

## Conclusions

In summary, this study presents a metabolic view on menopause. Post-menopausal women show large changes in fatty acid *β*-oxidation, and phospholipid, hormone and amino acid metabolism compared to pre-menopausal women. These findings increase the current understanding of menopause-induced metabolic changes, and will be useful for investigating diseases associated with menopause and evaluating confounding factors in the field of metabolomics.

## Supporting Information

S1 Data Baseline data and metabolomics data from 115 pre-menopausal and post-menopausal women. (XLSX)

S1 Fig PCA score plots for discriminating pre-menopausal women, post-menopausal women and quality control. (DOC)

S2 Fig Permutation tests with extra 2000 iterations to obtain robust P values for the PLS-DA models. (DOC)
