# Supplementary material for: Plasma Metabolic Profiles in Women are Menopause Dependent
Source: PLoS One. 2015 Nov 18;10(11):e0141743. doi: 10.1371/journal.pone.0141743 (PMC4651324; doi:10.1371/journal.pone.0141743)
Supplement: S1 Fig — (DOC) [file pone.0141743.s002.doc]

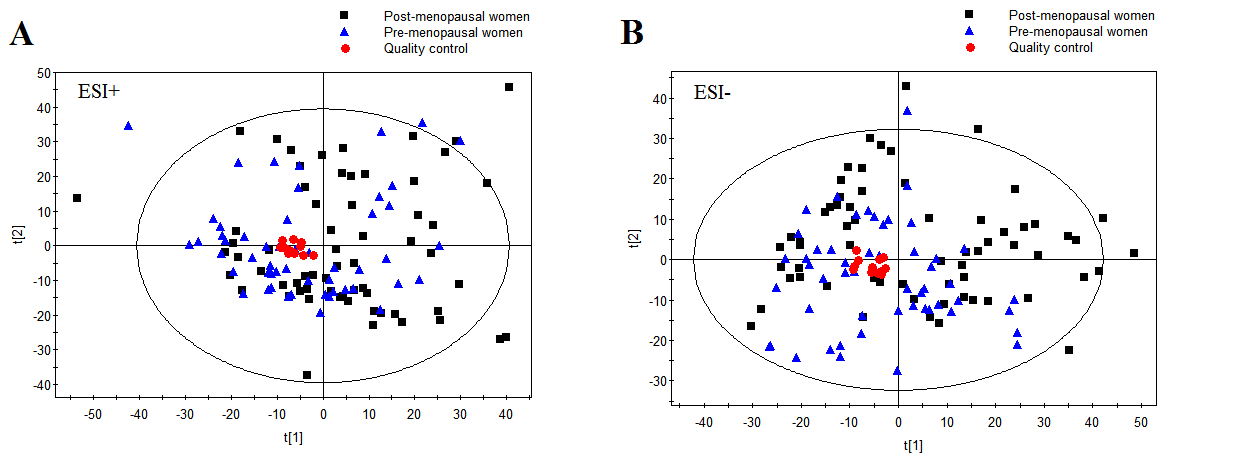


S1 Fig. (A) PCA score plot for discriminating pre-menopausal women, post-menopausal women and quality control in ESI+ mode. (B) PCA score plot for discriminating pre-menopausal women, post-menopausal women and quality control in ESI- mode.
