# Supplementary material for: Plasma Metabolic Profiles in Women are Menopause Dependent
Source: PLoS One. 2015 Nov 18;10(11):e0141743. doi: 10.1371/journal.pone.0141743 (PMC4651324; doi:10.1371/journal.pone.0141743)
Supplement: S2 Fig — (DOC) [file pone.0141743.s003.doc]

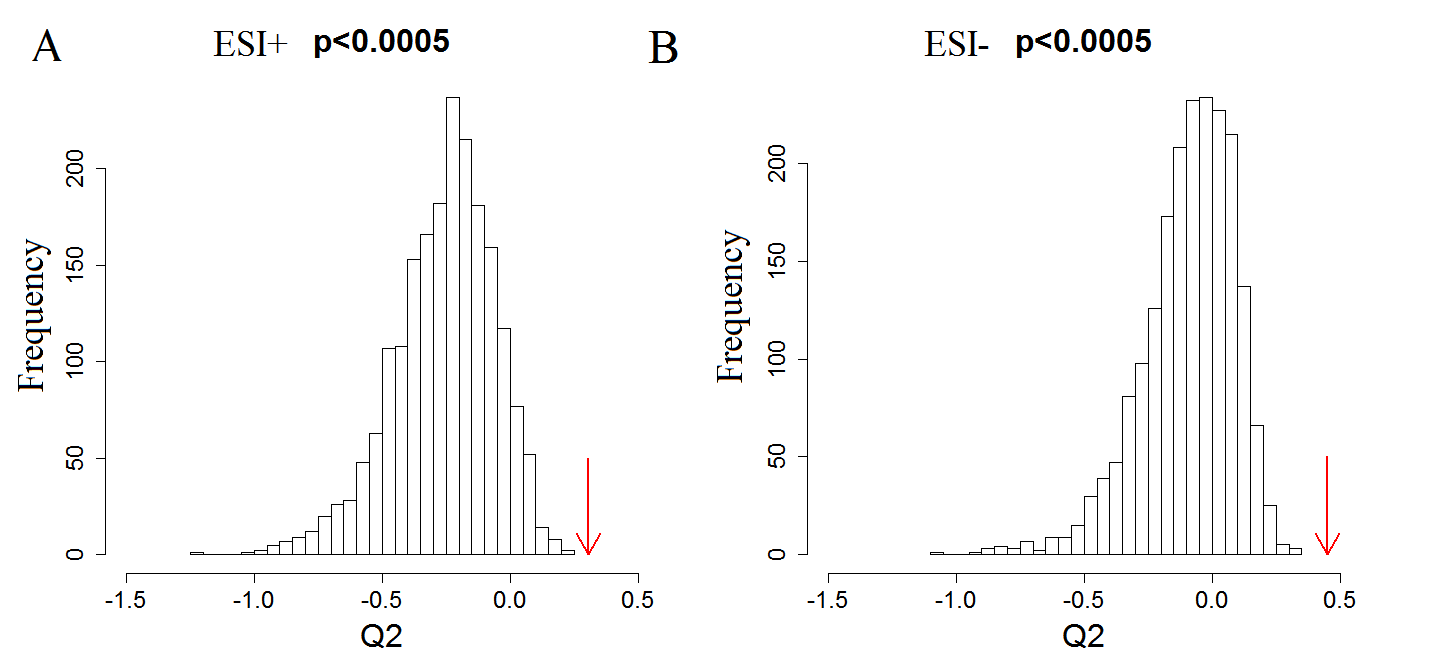


S2 Fig. Permutation tests with extra 2000 iterations were run to obtain robust P values for the PLS-DA models. (A) Permutation tests with 2000 iterations for pre-menopausal women *versus* post-menopausal women in ESI+ mode. (B) Permutation tests with 2000 iterations for pre-menopausal women *versus* post-menopausal women in ESI- mode.
